# Smoking Cessation Carries a Short-Term Rising Risk for Newly Diagnosed Diabetes Mellitus Independently of Weight Gain: A 6-Year Retrospective Cohort Study

**DOI:** 10.1155/2016/3961756

**Published:** 2016-07-12

**Authors:** Yi-Ting Sung, Cheng-Ting Hsiao, I-Jen Chang, Yu-Chih Lin, Chen-Yu Yueh

**Affiliations:** ^1^Department of Family Medicine, Chang Gung Memorial Hospital, Putz, Chiayi 613, Taiwan; ^2^Department of Emergency Medicine, Chang Gung Memorial Hospital, Putz, Chiayi 613, Taiwan; ^3^Chang Gung University of Science and Technology, Putz, Chiayi 613, Taiwan

## Abstract

*Background.* The effects of smoking on human metabolism are complex. Although smoking increases risk for diabetes mellitus, smoking cessation was also reported to be associated with weight gain and incident diabetes mellitus. We therefore conducted this study to clarify the association between smoking status and newly diagnosed diabetes mellitus.* Methods*. An analysis was done using the data of a mass health examination performed annually in an industrial park from 2007 to 2013. The association between smoking status and newly diagnosed diabetes mellitus was analyzed with adjustment for weight gain and other potential confounders.* Results.* Compared with never-smokers, not only current smokers but also ex-smokers in their first two years of abstinence had higher odds ratios (ORs) for newly diagnosed diabetes mellitus (never-smokers 3.6%, OR as 1; current smokers 5.5%, OR = 1.499, 95% CI = 1.147–1.960, and *p* = 0.003; ex-smokers in their first year of abstinence 7.5%, OR = 1.829, 95% CI = 0.906–3.694, and *p* = 0.092; and ex-smokers in their second year of abstinence 9.0%, OR = 2.020, 95% CI = 1.031–3.955, and *p* = 0.040).* Conclusion.* Smoking cessation generally decreased risk for newly diagnosed diabetes mellitus. However, increased odds were seen within the first 2 years of abstinence independently of weight gain.

## 1. Introduction

Smoking is the leading avoidable cause of premature death [1–5]. Cessation of tobacco use undoubtedly benefits health. However, many studies have reported that smoking cessation may implicate some hazard effects on health. It may sometimes cause weight gain and result in obesity [6–13], the second important preventable risk for premature death [14]. It may also increase diabetes mellitus risk in the short-term, presumptively owing to associated weight gain [13, 15]. But there are controversies among studies regarding smoking cessation, weight gain, and risk of incident diabetes mellitus. Baum and Chou reported in their NBER study that smokers were 7.8% less likely to be obese. The declining use of cigarettes was the most significantly attributing factor for the soaring prevalence of obesity in USA [16]. The average weight gain after smoking cessation varied widely and was roughly estimated to be around 4-5 kg in two large-scale studies [12, 17], approximately equal to the amount different between the mean weight of smokers and nonsmokers. However, Weitzman et al. reported that environmental exposure to tobacco smoke or active smoking in American adolescents was associated with higher rate of metabolic syndrome and abdominal obesity [18]. In Williamson et al.'s national cohort study, people who never smoked and smokers weighed nearly the same at a 7 to 13 years' follow-up. Marked weight gain (i.e., greater than 13 kg) may sometimes be strongly associated with smoking cessation, but it usually occurs in a minority of smoking quitters (i.e., in Williamson et al.'s study, 9.8 percent of the men and 13.4 percent of the women who quit smoking) [12].

Additionally, there are debates about the risk for incident diabetes mellitus following smoking cessation, although the association between smoking and diabetes mellitus has been well established [13, 19–24]. In this regard, Willi et al. did a meta-analysis on 25 prospective cohort studies including 1.2 million participants, with 45844 incidental cases of diabetes mellitus during a study follow-up period ranging from 5 to 30 years [19]. Compared with people who never smoked, the relative risk (RR) for diabetes mellitus in smokers was pooled and adjusted to be 1.44 (95% CI = 1.31–1.58). The risk was highest in heavy smokers (more than 19 cigarettes a day; RR = 1.61, 95% CI = 1.43–1.80) and lower in former active smokers (RR = 1.23, 95% CI = 1.14–1.33), consistent with a dose-response phenomenon. However, several studies disclosed controversial results. In Nagaya et al.'s longitudinal study, the risk for diabetes mellitus was increased by heavy smoking in obese men but decreased by light smoking in lean men [25]. On the other hand, study of Nakanishi et al. told another story. They found that smoking may dose-dependently increase risk for incident diabetes mellitus. But the relative risk was stronger in men with lower body mass index (body mass index less than 24.2 kg/m^2^ versus body mass index (BMI) of 24.2 kg/m^2^ or more) [26]. Nevertheless, Oba et al. suggested that “smoking cessation increases short-term risk of type 2 diabetes irrespective of weight gain” [27]. Likewise, Yeh et al.'s prospective cohort study found that the hazard for incident diabetes mellitus after smoking cessation reached its peak during the first 3 years (hazard ratio = 1.91; 95% CI = 1.19–3.05) and then gradually decreased to 0 at 12 years [15]. Furthermore, Kamaura et al. reported that smoking cessation only raised the rate of BMI increase briefly. There was even no increase in incidence of impaired fasting glucose [28]. Importantly, a prospective cohort study using the data from the Framingham Offspring Study disclosed that the cardiovascular benefit of smoking cessation was weakened by the presence of diabetes mellitus. But it was not influenced by subsequent weight gain [29]. Therefore, the occurrence of diabetes mellitus rather than weight gain following smoking cessation is the critical issue for care providers to encourage their clients abstaining from smoking.

Taking all the above together, it is crucial to clarify whether smoking cessation may indeed bring individuals harmful metabolic effects (i.e., sustained overweight or obesity and incident diabetes mellitus) and identify individuals vulnerable to its adverse effects. In this regard, there are fairly few studies exploring smoking cessation, incident diabetes mellitus, and weight change together. We therefore conducted this study to examine the association between smoking cessation and incident diabetes mellitus and its correlation with weight gain.

## 2. Materials and Methods

This retrospective cohort study was done after being approved by Chang Gung Memorial Hospital Institutional Review Board (Document number IRB 102-1014B). The profiles for analysis were extracted from the mass health examination performed for employees in an industrial park in middle Taiwan annually from 2007 to 2013. Self-reported smoking status (including how long they have smoked or quitted smoking), drinking habit, and medical history (including diseases such as diabetes mellitus, hypertension, dyslipidemia, and viral hepatitis and medication currently prescribed) were recorded by standardized questionnaires and were reconfirmed by a nurse-administered check-up. A total of 11032 people were included for screening. The female employees (455 in number) were excluded because they counted less than 5% of the total number, and all did not smoke ever. To avoid complexity, people (2125 in number) who did not have complete data, smoked just socially or resumed smoking during the study period, or were diagnosed as DM at first examination were all excluded as well. There were 8452 male employees eventually included in this study. All the included individuals were categorized into never-smokers who had never smoked before, ex-smokers who had quitted smoking, and current smokers who had been smoking until the final health examination. The ex-smokers were further divided by the number of years they had quitted smoking. The odds ratios (ORs) for newly diagnosed diabetes mellitus were then calculated with adjustment for potential confounders and compared among groups.

### 2.1. Laboratory Measurement

All biochemical tests were performed with fresh samples as instructed by manufacturer (7600 Clinical Analyzer, Hitachi High-Tech, Tokyo, Japan) under standardized quality control in the Clinical Laboratory Department at Chang Gung Memorial Hospital at Chiayi, Taiwan.

### 2.2. Definition of Diabetes Mellitus and Newly Diagnosed Diabetes Mellitus

The confirmation of diabetes mellitus usually needs two separated occasions of elevated plasma glucose higher than 6.9 mmol/L (125 mg/dL) or even strictly meeting the requirement of standard oral glucose tolerance test. In this retrospective study, it is hard to use these criteria. To avoid missing potential cases, people without history of diabetes mellitus but with just one occasion of fasting plasma glucose higher than 6.9 mmol/L (125 mg/dL) or newly receiving drug therapy for hyperglycemia were regarded as newly diagnosed diabetes mellitus.

### 2.3. Definition of Other Variables in the Analysis

The status of alcohol consumption was categorized as “drinks often,” “drinks occasionally,” and “drinks seldom.” People who just drank less than twice a month were regarded as “drinks occasionally.” Those who drank twice a month or more frequently were regarded as “drinks often.” Man with waist circumference 90 cm or higher was regarded as abdominal obesity. Systolic blood pressure equal to or higher than 130 mmHg, or diastolic blood pressure equal to or higher than 85 mmHg, or current use of antihypertensive medicines was regarded as high blood pressure. Serum triglyceride equal to or higher than 1.7 mmol/L (150 mg/dL) was regarded as dyslipidemia. Fasting plasma glucose from 5.6 to 6.9 mmol/L (100 to 125 mg/dL) was regarded as impaired fasting glucose.

### 2.4. Statistical Analysis

Logistic regression in SPSS 18.0 for Windows (SPSS Inc., Chicago, IL, USA) was used to estimate the ORs for newly diagnosed diabetes mellitus with smoking status as the main independent variable. The analysis was performed with adjustment for potential confounders including age, status of alcohol consumption, abdominal obesity, high blood pressure, dyslipidemia and impaired fasting glucose at the first examination, and weight gain between the first and the final examination. A *p* value less than 0.05 and a 95% confidence interval (CI) of OR not containing 1 were considered statistically significant. The numbers of mean are presented with standard deviations in parenthesis.

## 3. Results

The study included 8452 men aged, at their first health examination, from 24 to 70 with mean age 41.4 (±7.1) years. There were 4370 (51.7%) men who never smoked, 1169 (13.7%) men who had quitted smoking, and 2913 (34.5%) men who kept on smoking. In the group that had quitted smoking, there were 146 (1.7%) men within the first year of smoking cessation, 144 (1.7%) within the 2nd, 278 (3.3%) within the 3rd, and 202 (2.4%) within the 4th and 399 (4.7%) had quitted smoking longer than 4 years. The characteristics of the grouped individuals by smoking status were summarized in Table 1.

**Figure 1:**
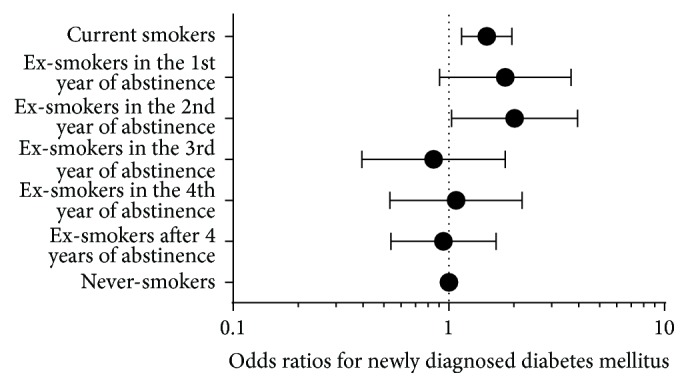
The comparison of the odds ratios for the incidence of newly diagnosed diabetes mellitus by smoking status. With never-smokers as reference (OR = 1), current smokers and ex-smokers in the first and second year of abstinence were inclined to have newly diagnosed diabetes mellitus, though the increased odds ratio for ex-smokers in the first year of abstinence was not statistically significant. All odds ratios were adjusted for age, alcohol consumption, abdominal obesity, high BP, dyslipidemia and impaired fasting glucose at their first examination, and weight gain between the first and the final examination.

During this 6-year period, 374 men (4.4%) were newly diagnosed as diabetes mellitus. Compared with never-smokers, the current smokers had higher odds to have newly diagnosed diabetes mellitus (as shown in Figure 1). Additionally, the ex-smokers within their first 2 years of abstinence were also inclined to have newly diagnosed diabetes mellitus (never-smokers 3.6%, OR as 1; current smokers 5.5%, OR = 1.499, 95% CI = 1.147–1.960, and *p* = 0.003; ex-smokers in the first year of abstinence 7.5%, OR = 1.829, 95% CI = 0.906–3.694, and *p* = 0.092; ex-smokers in the second year of abstinence 9.0%, OR = 2.020, 95% CI = 1.031–3.955, and *p* = 0.040; ex-smokers in the third year of abstinence 2.9%, OR = 0.850, 95% CI = 0.396–1.826, and *p* = 0.677; ex-smokers in the fourth year of abstinence 5.0%, OR = 1.080, 95% CI = 0.533–2.187, and *p* = 0.831; and the ex-smokers after 4 years of smoking cessation 4.0%, OR = 0.945, 95% CI = 0.539–1.658, and *p* = 0.845).

## 4. Discussion

Tobacco smoking is a well established risk factor for many diseases, including several kinds of cancer [30–32] and cardiovascular and lung diseases. It raises the death rate in middle age by twofold to threefold [2, 4]. In particular, it may predispose to or is associated with type 2 diabetes mellitus [13, 19–25, 27, 33–36], which further contributes to the risk of cardiovascular diseases [37]. However, there are controversies about metabolic benefits from smoking cessation. In terms of smoking cessation, weight gain, and diabetes mellitus, many studies disclose that smoking and excess weight are often inversely related. However, this association seems to interact significantly with age. In MacKay et al.'s study, never-smokers or ex-smokers aged 16–24 years were not more likely to be overweight or obese than active smokers of the same age [11]. Although smoking cessation could be accompanied with a weight gain, most of it occurs during the first 6 months [38]. The usual average weight gain is around 4-5 kg. People at younger age (e.g., <55 years) and lower socioeconomic status and who used to smoke heavily (e.g., more than 25 cigarettes per day) or have history of binge eating are at risk for marked weight gain (i.e., more than 10 kg) [12, 39]. Moreover, in Clair et al.'s study, weight gain following smoking cessation did not influence its cardiovascular benefit unless there was a coexisting diabetes mellitus [29]. Even in patients with diabetes mellitus, smoking cessation may still reduce risk of premature death although it usually takes several years for effect [40]. It then becomes crucial to determine whether smoking cessation* per se* would increase risk for incidental diabetes mellitus despite weight gain or not.

In our study, we found that ex-smokers during their first two years of abstinence have even higher odds than current smokers for newly diagnosed diabetes mellitus, though the increased odds ratio for ex-smokers in the first year of abstinence was not statistically significant (as shown in Figure 1). This tendency is independent of weight gain. It seems incredulous that people should immediately face rising odds for incident diabetes mellitus when they start quitting smoking. This contradictory result may arise from the immediate withdrawal of beneficial metabolic effect of certain constituents (e.g., nicotine) in tobacco [41–45] and the delayed subsiding of adverse effects or irreversible harmful effects from smoking [46, 47]. Like some diet drugs (e.g., sibutramine, phentermine, and buspirone), nicotine may suppress appetite and prevent weight gain by increasing central nervous system levels of norepinephrine, dopamine, and serotonin [48]. Smoking may influence appetite partially through the activation of hypothalamic melanocortin system [49]. It may also promote release of catecholamines and cortisol and suppress adiponectin [50, 51]. Moreover, the exposure to nicotine may increase beta cell apoptosis [52]. Although smoking is generally implicated with harmful effects, there are several studies that disclosed beneficial metabolic effects from certain constituents of tobacco [42–45]. Complexity of all the above findings could partially explain why smokers are prone to develop insulin resistance and have higher cardiovascular risk and the controversy why smoking cessation may sometimes not only cause weight gain [9, 53] but also increase incidence of diabetes mellitus [27, 28, 54].

There are a number of limitations in our study. The reasons people quitted smoking could not be explored. In particular, some of the ex-smokers might quit smoking because of great ill health. It may make us overestimate the smoking associated risk for incident diabetes mellitus. Additionally, the smoking status could not be further categorized by quantity of exposure. Given that smoking may reflect a clustering of risky life styles, there should be quite a few residual confounders in this study. In particular, diagnostic bias defining diabetes mellitus by only one occasion of abnormal fasting glucose, monogender, and the lack of counting the quantity of cigarette smoking all make the results of our study biased and of limitation. However, it may still provide some useful information. It discloses that smoking cessation generally decreases risk for incidental diabetes mellitus. But it may meanwhile carry a short-term (i.e., within the first couple of years) rising risk for incident diabetes mellitus. This association is independent of weight change.

## 5. Conclusion

Smoking cessation generally tends to decrease the incidence of newly diagnosed diabetes mellitus. However, rising odds are seen in the first 2 years after quitting smoking in our study. In particular, it is independent of weight gain. Therefore, we suggest that intensified modification of life style or other strategies for prevention of diabetes mellitus may be needed before and immediately after smoking cessation. At least, for smokers and ex-smokers at risk for diabetes mellitus, monitoring at shorter intervals should be considered for early detection.

## Figures and Tables

**Table 1:** Demographics of the studied individuals by smoking status and metabolic characteristics.

Smoking status	Never-smokers	Ex-smokers in the 1st year of abstinence	Ex-smokers in the 2nd year of abstinence	Ex-smokers in the 3rd year of abstinence	Ex-smokers in the 4th year of abstinence	Ex-smokers after the 4th year of abstinence	Current smokers	Total
Number of people	4370	146	144	278	202	399	2913	8452
Age at the 1st exam.	41.3 (7.4)	43.0 (7.4)	45.0 (8.8)	41.6 (4.7)	43.7 (8.3)	42.3 (6.3)	40.9 (6.5)	41.4 (7.1)
Abdominal obesity at the 1st exam.	1080 (24.7)	39 (26.7)	43 (29.9)	73 (26.3)	52 (25.7)	115 (28.8)	923 (31.7)	2325 (27.5)
Dyslipidemia at the 1st exam.	1280 (29.3)	52 (35.6)	51 (35.4)	76 (27.3)	60 (29.7)	124 (31.1)	1227 (42.1)	2870 (34.0)
High blood pressure at the 1st exam.	3405 (77.9)	119 (81.5)	126 (87.5)	212 (76.3)	164 (81.2)	327 (82.0)	2285 (78.4)	6638 (78.5)
Impaired fasting glucose at the 1st exam.	1020 (23.3)	47 (32.2)	47 (32.6)	64 (23.0)	62 (30.7)	111 (27.8)	696 (23.9)	2047 (24.2)
Weight gain (kg) at the final exam.	1.08 (4.07)	2.41 (3.87)	0.99 (5.66)	1.28 (4.23)	0.91 (4.41)	1.07 (4.43)	1.33 (4.32)	1.19 (4.22)
Newly diagnosed DM	157 (3.6)	11 (7.5)	13 (9.0)	8 (2.9)	10 (5.0)	16 (4.0)	159 (5.5)	374 (4.4)

Data are number of people or mean. Percentage and standard deviation are shown in parenthesis. Continuous variables such as age and weight gain were calculated as means with standard deviation (SD) in parenthesis.
